# A Web-Based Tailored Intervention to Support Illness Management in Patients With an Acute Coronary Syndrome: Pilot Study

**DOI:** 10.2196/cardio.7342

**Published:** 2017-09-06

**Authors:** Sylvie Cossette, José Côté, Geneviève Rouleau, Marie Robitaille, Sonia Heppell, Tanya Mailhot, Guillaume Fontaine, Catherine Cournoyer, Marie-Pierre Gagnon, Maria-Cecilia Gallani, Jean-Francois Tanguay, Jocelyn Dupuis, Anil Nigam, Marie-Claude Guertin

**Affiliations:** ^1^ Montreal Heart Institute Research Center Montréal, QC Canada; ^2^ Faculty of Nursing Université de Montréal Montréal, QC Canada; ^3^ Research Center of the Centre Hospitalier de l’Université de Montréal Montréal, QC Canada; ^4^ Research Chair in Innovative Nursing Practices Montréal, QC Canada; ^5^ Faculty of Nursing Laval University Quebec City, QC Canada; ^6^ Montreal Heart Institute Montreal, QC Canada; ^7^ Faculty of Medicine Université de Montréal Montréal, QC Canada; ^8^ Montreal Health Innovations Coordinating Center Montréal, QC Canada

**Keywords:** nursing informatics, health behavior, self-care, acute coronary syndrome, pilot study

## Abstract

**Background:**

Illness management after an acute coronary syndrome (ACS) is crucial to prevent cardiac complications, to foster participation in a cardiac rehabilitation (CR) program, and to optimize recovery. Web-based tailored interventions have the potential to provide individualized information and counseling to optimize patient’s illness management after hospital discharge.

**Objective:**

We aimed to assess the feasibility and acceptability of a Web-based tailored intervention (TAVIE@COEUR) designed to improve illness management in patients hospitalized for an ACS. Illness management outcomes were operationalized by self-care, medication adherence, anxiety management, cardiac risk factors reduction, and enrollment in a CR program.

**Methods:**

This posttest pilot study was conducted with one group (N=30) of patients hospitalized for an ACS on the coronary care unit of a tertiary cardiology center. TAVIE@COEUR comprises three Web-based sessions, with a duration ranging from 10 to 45 min and is structured around an algorithm to allow the tailoring of the intervention to different pathways according to patients’ responses to questions. TAVIE@COEUR includes 90 pages, 85 videos, and 47 PDF documents divided across session 1 (S1), session 2 (S2), and session 3 (S3). These sessions concern self-care and self-observation skills related to medication-taking (S1), emotional control and problem-solving skills (S2), and social skills and interacting with health professionals (S3). Throughout the videos, a virtual nurse (providing the intervention virtually) guides the participants in the acquisition of self-care skills. Patients completed S1 of TAVIE@COEUR before hospital discharge and were asked to complete S2 and S3 within 2 weeks after discharge. Feasibility indicators were extracted from the TAVIE@COEUR system. Data regarding acceptability (satisfaction and appreciation of the platform) and preliminary effect (self-care, medication adherence, anxiety management, risk factor reduction, and CR enrollment) were assessed through questionnaires at 1 month following discharge. Preliminary effect was assessed by comparing baseline and 1-month illness management variables.

**Results:**

Of the 30 participants, 20 completed S1, 10 completed S2, and 5 completed S3. Good acceptability scores were observed for ease of navigation (mean=3.58, standard deviation [SD]=0.70; scale=0-4), ease of understanding (mean=3.46, SD=0.63; scale=0-4), and applicability (mean=3.55, SD=0.74; scale=0-4). The lowest acceptability scores were observed for information tailoring (mean=2.93, SD=0.68; scale=0-4) and individual relevance (mean=2.56, SD=0.96; scale=0-4). With regard to preliminary effect, we observed an overall self-care at 1 month following discharge score higher than at baseline (mean at 1 month=54.07, SD=3.99 vs mean at baseline=49.09, SD=6.92; scale-0-60).

**Conclusions:**

Although participants reported general satisfaction and appreciation of TAVIE@COEUR, acceptability and feasibility results show the need for further development of the Web-based intervention to enhance its tailoring before undertaking a full-fledged randomized controlled trial. This may be accomplished by optimizing the adaptability of TAVIE@COEUR to patients’ knowledge, needs, interests, individual capabilities, and emotional and cognitive responses during session completion.

## Introduction

Health behavior change and illness management play a critical role in the treatment for patients with an acute coronary syndrome (ACS) [1,2]. However, the short hospitalizations following an ACS leave little time for health professionals to provide tailored information and support to patients before discharge [3-6]. This often results in patient’s information needs not being met and, in turn, makes it challenging for them to engage in healthful behaviors and illness management [1,5,7-9]. Illness management is defined as a broad set of strategies and self-care–related behaviors that can be enacted by patients to optimize recovery, reduce their susceptibility to relapses, cope effectively with their symptoms, and manage the impacts of the illness of their quality of life [10]. In the context of cardiovascular diseases, many aspects of illness management such as self-care, medication adherence, anxiety management, cardiac risk factor reduction, and enrollment in a cardiac rehabilitation (CR) program are essential for optimizing recovery and preventing cardiac complications [1,5]. Medication adherence can reduce the risk of all-cause mortality by 35% [7]. Similarly, symptom management and reduction of cardiac risk factors, as promoted in CR programs, are positively linked to cardiac patient outcomes, including increased quality of life and lowered health care services utilization [1,11]. Anxiety management is vital after an ACS, since anxiety may modulate other risk factors such as depression, smoking, sedentary lifestyle, substance abuse, and overweight [12]. Moreover, anxiety is a negative and independent factor influencing adherence to treatment in patients with acute coronary syndrome [13]. Intervention efforts should then focus on engaging ACS patients in practicing self-care, being adherent to their medication, managing their anxiety and cardiac risk factors, and enrolling in a CR program [5,14].

Health professionals in clinical settings play a crucial role in helping patients initiate illness management and health behavior change [1,5]. Clinical practice guidelines clearly state that tailored counseling on the part of health professionals in primary health care settings is a key factor to help patients initiate such behaviors and reduce cardiovascular risk [15-18]. However, it is often reported that nurses and other health professionals neither have the time nor the resources to provide such information and coaching in a timely manner while the patient is hospitalized [19-22]. A recent study on the matter concluded that patient education requires advanced communication skills and pedagogical competences on the part of health professionals. Such skills and competences are needed to provide effective counseling that will result in patients initiating and maintaining illness management–related behaviors [19]. We must then consider that although it is essential to provide tailored counseling to patients following an ACS, only a few health professionals have the necessary knowledge and skills to do so.

Web-based interventions are promising avenues to enhance patient access to information resources post discharge [1,9,23]. Although the number of these technology-driven interventions is growing exponentially in the health care field [24], results on their efficacy remain unclear [1,9,23]. Indeed, some studies have shown that the use of information and communications technologies (ICTs) can positively affect illness management [25,26]. On the other hand, other authors argue that home Internet access, prior Internet experience, and engagement of patients are challenges that must be resolved for Web-based interventions to have a significant effect [27]. A systematic review examining mobile Web-based interventions for self-care related to medication intake showed significant improvement in medication adherence in 18 out of 29 studies [28]. Another systematic review on the same topic by Anglada-Martinez et al [29] showed positive outcomes in 65% of studies, with the authors emphasizing the need for more extensive research. Moreover, to our knowledge, only a few studies have assessed Web-based tailored interventions to improve illness management with ACS patients specifically.

Therefore, the purpose of this pilot study was to develop TAVIE@COEUR, a Web-based tailored intervention including videos showcasing a virtual nurse, and assess its acceptability, feasibility, and preliminary effect for improving illness management in patients hospitalized for an ACS.

## Methods

### Study Design and Setting

The pilot study used a posttest design with one group of ACS patients hospitalized on the Coronary Care Unit (CCU) in a tertiary cardiology center in Canada. The study was approved by the institutional review board of the Montreal Heart Institute Research Center (project number: 11-1320). Our study is reported in accordance with the CONSORT-EHEALTH checklist version 1.6.1. (see Multimedia Appendix 1) [30]. No content or methodological modifications were made after study commencement.

### Participants

We recruited a convenience sample of patients hospitalized at the CCU. To be included in the study, participants had to be aged >18 years, be hospitalized for an ACS, be discharged to home within 7 days, understand written and spoken French, and have the physical and cognitive abilities to participate. Patients were excluded if they were hospitalized on the CCU for more than 7 days because this would reflect a more difficult ACS recovery, potentially interfering with their physical abilities to participate.

### Procedure

Enrollment and follow-up occurred from June 2014 to August 2015. Participants were recruited during face-to-face encounters with the project nurse at the CCU. After receiving an explanation regarding the study and providing written consent, patients completed a baseline questionnaire. An individual identification number, username, and password were then provided to participants to allow them to log in to the TAVIE@COEUR Web-based platform. The project-dedicated nurse then showed participants how to use TAVIE@COEUR on a tablet computer and invited them to complete the first session (20 min) at the CCU. Participants were then asked to complete the next two sessions of TAVIE@COEUR at home within 2 weeks of discharge.

All patients received usual care during their hospitalization, including a predischarge teaching session by the bedside nurse on resuming activities of daily living, cardiovascular risk factors, and new medications. This included a pamphlet about medication, prevention of complications, local CR program, and postangioplasty care, which is provided to all patients discharged from the CCU following a coronary event.

### The TAVIE@COEUR Web-Based Tailored Intervention

**Figure 1:**
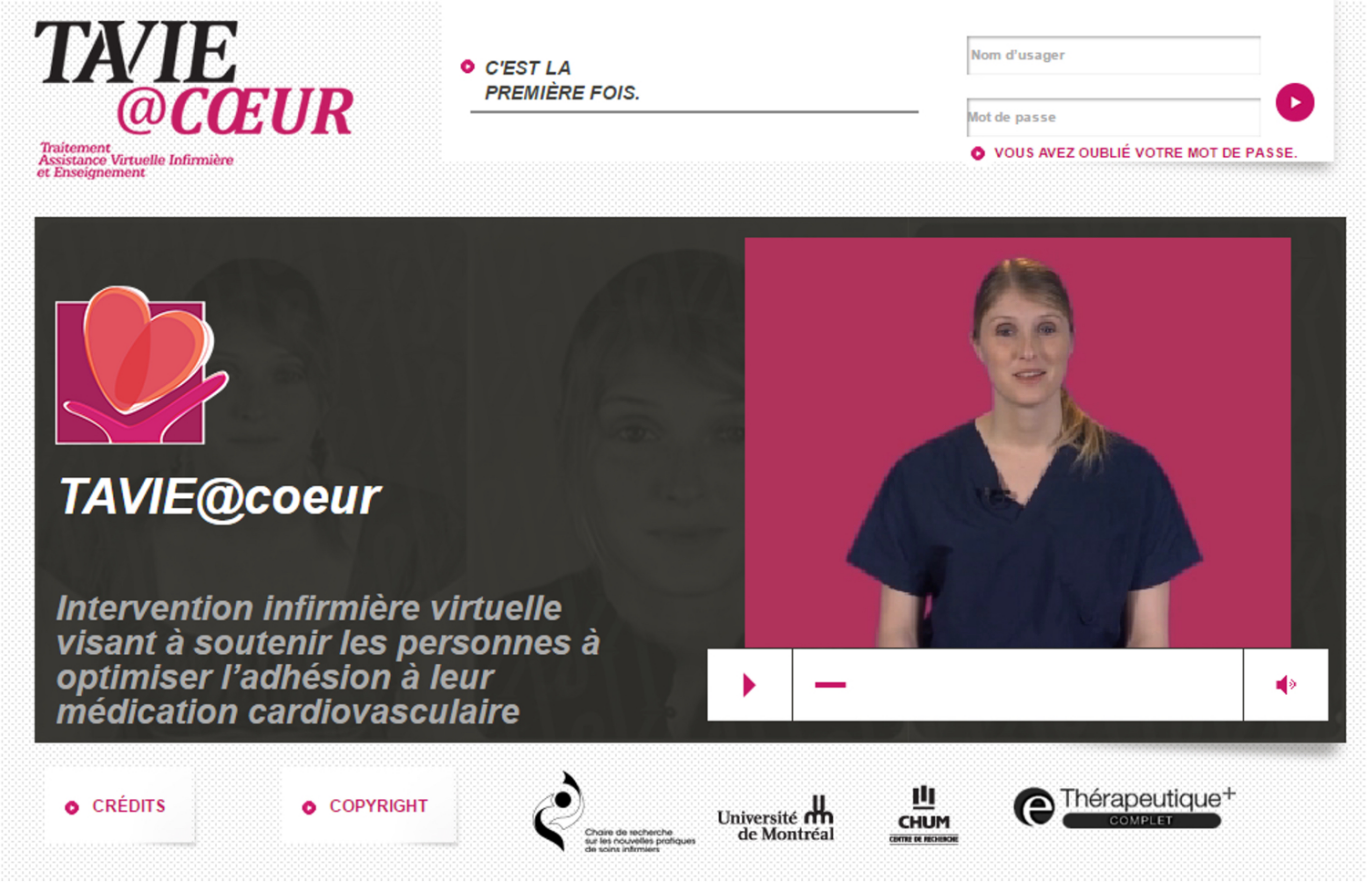
TAVIE@COEUR homepage and virtual nurse (in French).

TAVIE@COEUR (in French, which translates as YOURLIFE@HEART in English) is a Web-based tailored intervention, including videos in which a virtual nurse interacts with the patient. The ultimate goal of TAVIE@COEUR is to improve patient illness management by providing information and resources tailored to the needs of every patient, while being easily accessible from home. TAVIE@COEUR is completely asynchronous and led by a virtual nurse who guides patients through a learning process regarding the development of illness management skills for self-care and medication adherence (see Figure 1).

**Figure 2:**
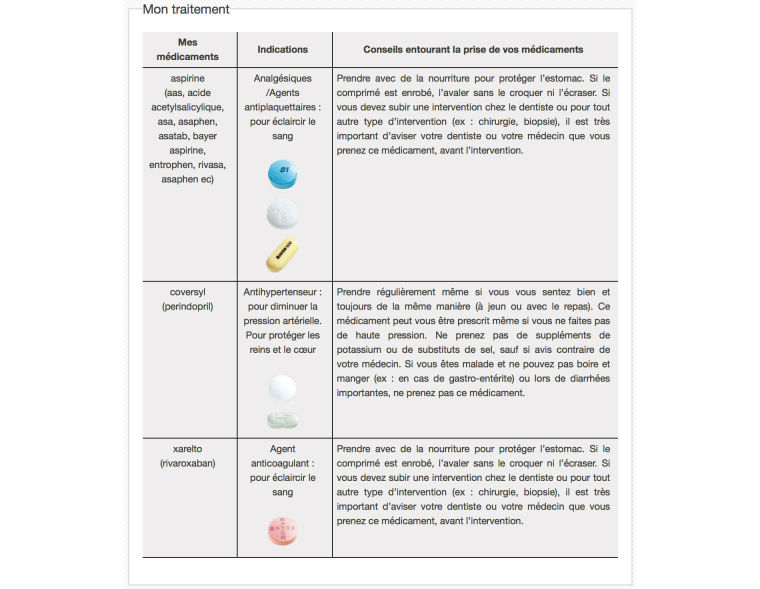
Example of specific information provided regarding the medication of each patient (generic and commercial names, indications and tips - in French).

#### Development Process

TAVIE@COEUR is based upon the TAVIE virtual nursing assistance and education Web platform developed by Côté et al [31]. The TAVIE platform was evaluated in different populations such as human immunodeficiency virus (HIV)-positive patients [32].

The TAVIE@COEUR platform was designed with an interdisciplinary team of researchers, nurse practitioners, managers, pharmacists, physicians, and computers scientists. This ensured that everybody involved would agree on the information and content of the platform. All professionals involved in the project team (nurses, pharmacists, and physicians) validated the content, including the information about medication, side effects, therapeutic goals, misuses of medication, and treatment. To ensure feasibility, the managers of the CCU validated the format of the TAVIE@COEUR platform, such as the duration and sequence of sessions. Computer scientists provided feedback on the structure and content of TAVIE@COEUR, as well as measures of acceptability.

To ensure the preservation of data related to TAVIE@COEUR, the website and data regarding its use were hosted on secure computer servers at the research setting.

#### TAVIE@COEUR Access

TAVIE@COEUR could be accessed via a fixed URL. Participants could log in to their account on their home computer or tablet computer using their personal log-in credentials created during their stay at the CCU.

#### TAVIE@COEUR Content

A general overview of the content of the three sessions of TAVIE@COEUR is presented in Table 1. The TAVIE@COEUR intervention is based on a motivational and self-efficacy enhancing framework. More specifically, the interventions of the virtual nurse throughout the 85 videos of TAVIE@COEUR are based on the beliefs and representations of individuals (self-regulation theory by Leventhal; [33]), integrate motivational counseling (works by Miller and Rollnick; [34]), and take into account the stages of change of Prochaska [35].

**Table 1:** General overview of the content of TAVIE@COEUR.

Sessions and general theme		Number of Web pages	Number of videos	Number of PDF documents
Home page		1	1	0
**Session 1: Self-care and self-observation skills**		17	14	35
	Session 1 objectives	1	1	0
	Taking your medication	3	3	0
	Identifying or finding out about your medication	2	0	0
	Getting motivated to take your medication	1	1	3
	Associating a positive image to your medication	3	3	0
	Observing your own behavior	1	2	0
	Identifying adverse effects or discomfort	1	1	2
	Managing adverse effects or discomfort	2	0	19
	Documenting adverse effects or discomfort	1	1	2
	Tips to avoid adverse effects or discomfort	1	1	0
	Integrating strategies for medication-taking	1	1	9
**Session 2: Emotional control and problem-solving skills**		31	28	7
	Session 2 objectives	1	1	0
	Assessing the efficacy of advice	1	0	0
	Managing adverse effects or discomfort	4	2	0
	Associating a positive image to your medication	6	5	0
	Observing your own behavior	3	2	0
	Managing challenges in medication-taking	3	5	0
	Recognizing and changing negative thoughts	2	4	1
	The DECIDE^a^approach	6	8	4
	Identifying or finding out about your medication	2	0	0
	Managing adverse effects or discomfort	2	0	0
	Documenting adverse effects or discomfort	1	1	2
**Session 3: Social skills and interacting with health professionals**		41	42	5
	Session 3 objectives	2	2	0
	Associating a positive image to your medication	9	7	0
	Observing your own behavior	6	4	0
	Managing negative feelings	6	7	0
	The DECIDE approach	1	1	0
	Recognizing the importance of support	2	2	1
	Listening and communication skills	3	3	1
	Resources, services, and health professionals	3	12	2
	Practicing the session strategies	2	1	1
	Managing adverse effects or discomfort	4	2	0
	Identifying or finding out about your medication	2	0	0
	Documenting adverse effects or discomfort	1	1	0
Total		90	85	47

^a^DECIDE is an acronym designed for helping patients solve problems. D=Describe the situation in which the oversight occurred to identify your difficulty; E=Express a list of possible strategies to address this challenge; C=Choose the strategy that is most likely to be effective and with which you feel comfortable; I=Imagine yourself using this strategy; D=Decide to take action and face the situation by putting this strategy into practice; E=Evaluate results and resume problem-solving skills when you are not satisfied with the results.

In session 1 (S1), the overarching goal is to develop self-care and self-observation skills. The virtual nurse invites the participant to identify and find out about his medication, while normalizing any possible omission of medication (see Figure 2). She also encourages the participant to continue with successful techniques to optimize medication adherence and recognize signs and symptoms of deterioration or medication side effects. The virtual nurse then suggests possible solutions to any problems in these areas. Session 2 (S2) focuses on emotional control and problem-solving skills. The session addresses possible difficult situations that may lead to nonadherence, for example, travel, restaurants, or activities that can interfere with the daily routine. Negative feelings toward medication, fear of side effects, and confusion about interaction between illicit drugs and prescribed medications are also discussed. Finally, session 3 (S3) focuses on developing patients’ social skills and interactions with family and health care professionals to strengthen their support system and to improve illness management.

#### TAVIE@COEUR Structure

TAVIE@COEUR comprises three Web-based sessions, each with a mean duration of 30 min. However, the duration of each session could vary between 10 and 45 min, depending on the needs of each patient and their answers during the navigation. TAVIE@COEUR includes 90 pages, 85 videos, and 47 PDF documents divided across S1, S2, and S3.

TAVIE@COEUR is based on an algorithm allowing a tailored intervention, meaning that throughout the intervention, participants are asked 46 questions (S1: 5 questions, S2: 16 questions, and S3: 25 questions) to tailor the information and resources to their individual needs. For instance, participants are asked to enter their prescription drug information, allowing tailored information to be provided on these specific medications throughout the 3 sessions of the intervention. An example of question asked is: “Do you take ONE of the following medications: Acetylsalicylic Acid, Clopidogrel, Prasugrel, or Ticagrelor?” If the patient answers yes, another question is asked: “During the past week, have you forgotten taking any of your antiplatelet medication?” If the patient answers yes again, a video is presented to provide specific information regarding the benefits of antiplatelet medication and motivational counseling regarding this subject. If the patient answers no to these questions, the intervention proceeds to the next subject. The tailoring of TAVIE@COEUR means that some pages are mandatory for all patients, whereas other pages appear depending on patients’ personal responses. Additionally, PDFs can be accessed to get more detailed information on subjects of interest for the patient.

#### Use Parameters

Participants completed S1 on tablet computers at the CCU with headphones. We asked participants to complete the two other sessions at home within 2 weeks of discharge.

#### Level of Human Involvement and Cointerventions

The Web-based tailored intervention was completely asynchronous. The research team was available to provide technical support via mail or telephone. No other intervention was performed during the study.

### Measures

#### Sociodemographic and Clinical Data

Baseline sociodemographic and clinical variables were collected for all participants. Some variables were obtained from medical charts such as age, gender, medical diagnosis, and both cardiovascular and noncardiovascular antecedents. Other variables were self-reported, including employment status, education, marital status, and place of residence.

#### TAVIE@COEUR Feasibility

Feasibility determines “whether the intervention, study design, and procedures can be successfully executed by the researcher and delivered to the participants as planned” [36]. The indicators of feasibility of TAVIE@COEUR were the following: (1) the number of patients who browsed each session, (2) the number of patients who completed each session, and (3) the duration of each session for those who completed it.

#### TAVIE@COEUR Acceptability

Acceptability determines “the suitability of the intervention and the study procedures from the perspective of the clinical population of interest, the intervention providers, or the health professionals who provide care to the population of interest” [36]. The acceptability of TAVIE@COEUR was measured by the Web-based Nursing Intervention Acceptability Scale developed by Côté et al [32]. The scale includes 21 items assessing satisfaction in 8 dimensions: ease of navigation, ease of understanding, credibility, tailoring of information, individual pertinence, applicability, appreciation of user interface design, and general appreciation. Patients indicated the degree to which they agreed with each statement from *not at all* (0) to *totally* (4). The score for each dimension was calculated by summing the scores for the items therein and dividing the result by the number of items in the dimension to standardize the reported means and allow comparison between dimensions. A higher total score for each dimension indicates greater acceptability (possible range: 0-4).

#### TAVIE@COEUR Preliminary Effect

Preliminary effect measures in illness management (self-care, medication adherence, anxiety management, cardiac risk factors reduction, and CR enrollment) were assessed at baseline and at 1 month after discharge by telephone. Enrollment in a CR program, hospitalizations, and emergency visits were assessed at 1 and 3 months.

Self-care was assessed with the Therapeutic Self-Care Scale (TSCS) that measures actions taken by a patient to promote, maintain, or improve health; prevent sickness; detect and manage symptoms; and regain normal functioning [37]. Patients indicated whether they agreed with each statement from *not at all* (0) to *totally* (5), with higher total scores indicating better self-care reported abilities (possible range: 0-60). Cronbach alpha coefficients for the total score were ranged between .88 and .93 [38]. We also used the three subscores proposed by Chaboyer et al [39], who conducted a principal component analysis. The three subscores included perceived capabilities of *taking medications* (alpha: .80 and .79 for the 3- and 6-months data, respectively, in their study), *recognizing and managing symptoms* (alpha: .71 and .70 for the 3- and 6-months data, respectively), and *managing changes in health conditions* (alpha: .48 and .50, for the 3- and 6-months data, respectively) [39].

Medication adherence was assessed with the Morisky Self-Reported Medication-Taking Scale (SRMTS) [40] that assesses reasons for nonadherence. Patients indicated whether (1) or not (0) they forgot, omitted, were careless, or stopped their medication when feeling better at 30 days after discharge (possible range: 0-4). A total score of zero (0) represents no omission of medication, reflecting high adherence; a score of 1 or 2, medium adherence; and a score of 3 or 4, low adherence [40]. Morisky et al [40] reported a Cronbach alpha of .61.

Anxiety was assessed with the State-Trait Anxiety Inventory–State version [41] that includes negatively-worded and positively-worded items. Patients indicated whether they agreed with each statement from “not at all” (1) to “a lot” (4). Higher scores indicate higher anxiety levels (possible range: 20-80). Spielberger et al [41] reported internal consistency coefficients ranging from .86 to .95 and test-retest reliability coefficients ranging from .65 to .75 over 2 months [42].

Cardiac risk factors were assessed using the questionnaire *Do you have a healthy heart?* [43] that measures the following risk factors: age, gender, menopausal status, heredity, physical exercise, smoking, waist size, weight, diabetes, arterial tension, as well as levels of cholesterol (total, high- and low-density lipoprotein) and of triglycerides. Each item is rated differently. For instance, for physical activity, the following question is asked: “In general, how many days per week are you physically active for at least 30 minutes (walking, dancing, sports, workout, etc; does not have to be a continuous 30 minutes).” Possible answers include less than once a week, 1 to 2 times per week, and 3 to 4 times per week. Scores for each item were added to obtain a total score representing a patient’s level of risk; higher scores representing a higher level of risk (possible range: 0-144). No studies have yet established the validity of the scale, which was created by experts for clinical purposes. However, in a previous study, the Cronbach alpha was .71 [44].

Information on enrollment in a local CR program, hospitalizations, and emergency room visits was collected from the electronic medical charts of the research hospital.

#### Sample Size

In accordance with pilot study guidelines, we targeted a sample size of 30 participants [45,46]. No power analyses were performed in the context of a pilot study.

### Statistical Analysis

Descriptive statistics such as means and standard deviations (SDs) were used for continuous variables, whereas count and percentage were used for dichotomous variables. As this was a pilot study, no inferential analyses were planned; preliminary effect results being provided strictly for illustrating general trends as recommended in pilot study methodology [46].

## Results

### Characteristics of the Sample

Participants (N=30) were enrolled in the study from May 2014 to June 2015. Most participants were men, with a mean age of 59 years (SD 9). The majority of the participants were born in Canada and living with a partner (see Table 2).

**Table 2:** Participants’ baseline sociodemographic and clinical data (N=30).

Characteristic		n (%)
Sex (male)		26 (87)
Place of birth (Canada)		24 (80)
Living situation (with a partner)		23 (77)
Employment status (employed)		16 (53)
Education (high school or higher)		15 (50)
Medical antecedents (≥1)		20 (67)
Cardiovascular antecedents (≥1)		11 (37)
**Medical diagnostic**		
	Myocardial infarction (STEMI)	18 (60)
	Myocardial infarction (NSTEMI)	7 (23)
	Unstable angina pectoris	5 (17)
**Cardiovascular risk factors**		
	Alcohol use	19 (63)
	Hypertension	16 (53)
	Hypercholesterolemia	15 (50)
	Physical inactivity	15 (50)
	Stress	15 (50)
	Family history of cardiovascular disease	14 (47)
	Smoking	13 (43)
	Obesity	10 (33)

### TAVIE@COEUR Feasibility

As shown in Table 3, S1, S2, and S3 were browsed by 30, 17, and 10 participants, respectively. Of these participants, 20 out of 30 completed the mandatory pages in S1, 10 participants completed the mandatory pages in S2, and 5 participants completed the mandatory pages in S3. For those who completed the mandatory pages, the first session lasted a mean of 25 min, whereas the second and third sessions lasted around 16 min.

### TAVIE@COEUR Acceptability

The general level of satisfaction toward TAVIE@COEUR stayed mostly the same following S1 and following either S2, S3, or both (see Table 4). Items that scored the highest on the acceptability scale were related to the ease of navigation throughout TAVIE@COEUR and the ease of understanding the textual and video content of TAVIE@COEUR. Items that scored the lowest were related to the tailoring of the information and the individual relevance of content in TAVIE@COEUR. Patients identified other areas for improvement such as the pacing of the intervention by the virtual nurse, which they felt was not tailored to their needs. Some believed the content to be less suitable for people who already had a good knowledge of disease and drugs, whereas other patients would have preferred more information on how to prevent the recurrence of cardiac events.

Certain aspects of the intervention scored higher after S1 (tailoring of information and individual relevance), whereas other factors pertaining to the interface scored higher after S3 (ease of navigation and user interface design).

**Table 3:** Feasibility of TAVIE@COEUR (N=30).

Feasibility outcomes		Mean (SD^a^) or n (%)
**Session 1**		
	Participants who began the session, n (%)	30 (100)
	Participants who completed the session, n (%)	20 (67)
	Mean duration of session if completed, in minutes, mean (SD)	25 (9)
**Session 2**		
	Participants who began the session, n (%)	17 (57)
	Participants who completed the session, n (%)	10 (33)
	Mean duration of session if completed, in minutes, mean (SD)	16 (7)
**Session 3**		
	Participants who began the session, n (%)	10 (33)
	Participants who completed the session, n (%)	5 (17)
	Mean duration of session if completed, in minutes, mean (SD)	16 (5)

^a^SD: standard deviation.

**Table 4:** TAVIE@COEUR acceptability (n=26).

Outcome variable	Number of items	Possible range	Standardized mean (SD^a^) score after session 1	Standardized mean (SD) score after sessions 2, session 3 or both
Ease of navigation	2	0-4	3.35 (0.74)	3.58 (0.70)
Ease of understanding	2	0-4	3.43 (0.78)	3.46 (0.63)
Credibility of the information	1	0-4	2.91 (0.92)	3.27 (0.70)
Tailoring of the information	4	0-4	3.26 (0.57)	2.93 (0.68)
Individual relevance	4	0-4	2.97 (0.73)	2.56 (0.96)
Applicability	1	0-4	3.09 (0.87)	3.55 (0.74)
Appreciation of user interface	5	0-4	2.94 (0.67)	3.04 (0.74)
General appreciation	2	0-4	3.27 (0.72)	3.24 (0.87)
General satisfaction score	21	0-4	3.14 (0.57)	3.02 (0.61)

^a^SD: standard deviation.

### TAVIE@COEUR Preliminary Effect

We observed that an overall self-care score 1 month post discharge was higher than the baseline score (see Table 5). Moreover, post-intervention scores for the three dimensions of self-care (medication-taking, recognizing and managing symptoms, and managing changes in the health condition) were all higher than baseline scores.

Responses on the medication adherence scale were skewed toward the score of 0: half of patients reported no omission (score of 0) of medication at 1 month after discharge. Therefore, patients were classified as either reporting no omission (score of 0) versus scores ≥1 omissions. When examining each of the four items of the SRMTS scale, the main reason for nonadherence was *forgetting* (item 1) to take medication. Seven percent (2 out of 27) of patients scored on either items 2 to 4 on *omission*, *being careless*, and *stopping medication when feeling better*. Patients generally reported a low anxiety level at 1 month post discharge.

**Table 5:** TAVIE@COEUR preliminary effect.

Outcome variable		Number of items	Possible range	Mean (SD^a^) score at baseline (n=30)	Mean (SD) score or n (%) at 1 month (n=27)
**Overall self-care**		12	0-60	49.09 (6.92)	54.07 (3.99)
	Self-care related to medication-taking	3	0-15	11.35 (2.41)	13.74 (1.32)
	Self-care related to recognizing and managing symptoms	4	0-20	15.69 (3.49)	16.86 (2.36)
	Self-care related to managing changes in health condition	3	0-15	13.24 (2.29)	14.19 (1.47)
Medication adherence (no omission), n (%)		NA	NA	NA	14 (52)
Anxiety		20	20-80	NA	33.36 (12.28)
Cardiac risk factors		25	0-144	NA	83.96 (11.25)
Enrollment in cardiac rehabilitation, n (%)		NA	NA	NA	12 (40)

^a^SD: standard deviation.

## Discussion

### Principal Findings

We developed and pilot-tested TAVIE@COEUR, a Web-based tailored intervention aimed at supporting illness management in patients hospitalized for an ACS. Feasibility and acceptability results suggest some strengths such as the ease of navigation and the content of TAVIE@COEUR but also underline the need for further development of TAVIE@COEUR. Indeed, we believe that further tailoring of TAVIE@COEUR should be done before undertaking a full-fledged randomized controlled trial (RCT). This may be necessary to increase the engagement of participants to complete all sessions, possibly by interchanging some concrete and more abstract content across the sessions. Preliminary effect results are promising, with improved self-care scores 1 month post discharge related to medication-taking, management of symptoms, and management of changes in the health condition.

Although acceptability scores regarding the TAVIE@COEUR Web-based tailored intervention were generally good, the low completion rate of the 3 sessions underline the significant challenges related to optimizing engagement and adherence in Web-based interventions for illness management. Participants appreciated the ease of navigation throughout the Web-based platform, the ease of understanding of the textual and multimedia content, and the applicability of the information in TAVIE@COEUR. However, relatively low scores were observed for other dimensions of the acceptability questionnaire, such as individual relevance and tailoring of the information; some participants felt that TAVIE@COEUR was not tailored enough to their needs. Moreover, feasibility was not optimal since the global participation rate in the Web-based intervention decreased over the three sessions: 20 out of 30 participants completed S1 (at the CCU), 10 completed S2 (at home), and 5 completed S3 (at home). The lower-than-normal participation rate in the second and third sessions of TAVIE@COEUR may be explained by a variety of factors, including lower motivation to continue the intervention up to the end and lack of individual relevance of the intervention content in the third session. Indeed, it is possible that the content of S3, focusing on social skills and interactions with health professionals, was seen less relevant to participants’ needs, especially because they received all crucial information on medication adherence during the first two sessions. However, the mean duration of sessions was consistent throughout the intervention, suggesting that participants who completed S3 were similarly engaged in the intervention.

More and more electronic health (eHealth) apps and Web-based interventions are addressing health behavior change and are being evaluated in patients with various chronic illnesses [47-49]. Patients’ engagement in completing Web-based interventions is often reported in eHealth intervention research as being about 50% of patients recruited [50,51]. This means that dropout rates of around 50% are observed in several Web-based interventions, limiting their effectiveness in initiating and maintaining changes in health behaviors in patients with cardiovascular diseases [47,48,51-54]. This phenomenon is explained at least, in part, by the fact that most existing Web-based interventions, even those that are called *individualized*, do not take into account the navigation behavior, knowledge, preferences, and individual objectives of the real-time users [51].

We believe the true challenge lies in developing new strategies for engaging participants with various information needs and individual capabilities in Web-based interventions, such as TAVIE@COEUR. In this study, tailoring was ensured by participants following their own paths, choosing to skip or view the videos, and selecting the appropriate resources to match their needs. However, the general theme of the intervention (illness management geared toward medication-taking) was similar for all participants. We observed that, whereas all participants went through the same life-threatening coronary event and associated medical treatment, their information needs varied widely, more than anticipated with the tailoring allowed in TAVIE@COEUR. Some participants felt the content of TAVIE@COEUR provided more than enough information for their needs, whereas other patients would have liked different resources not related to medication-taking because they were already accustomed to taking medication regularly.

The findings of this study suggest the need for more variety in the options offered to each individual to better match the individual needs of each patient [55,56]. For instance, intelligent tutoring systems (ITS) show great promise to provide tailored resource to patients by adapting the structure and content of the training to learners’ knowledge, preferences, and individual capabilities by using some components of artificial intelligence [57]. ITS are generally conceptualized using four models: (1) the interface model, by which the participant interacts with the ITS; (2) the domain model, which represents the knowledge to learn (clinical and theoretical content); (3) the learner model, which takes into account the knowledge, preferences, and individual capabilities of each participant; and (4) the tutor model representing the pedagogical strategies used throughout the ITS [58]. The combination of these four models, through the use of machine learning and data mining, allow for the tailoring of the instruction to each participant [58]. Whereas ITS with various levels of complexity have been evaluated in academic settings with positive results, very few ITS have been assessed with patients in health care settings [59,60]. We can hypothesize that user-modeling at the beginning of TAVIE@COEUR could allow to better target patients with illness management problems related to medication-taking and redirect those who are okay to other resources related, for example, to nutritional guidelines or ACS pathophysiology. In this sense, we believe a widest range of content should be developed to better represent the interests and needs of patients affected by cardiac diseases.

Future research should also focus on developing new strategies for engaging participants in Web-based interventions, such as interactive feedback based on real-time emotional and cognitive responses, online support groups, serious games, and blended Web-based or in-person interventions. Increasing the accessibility of such interventions is also an important challenge; researchers should make sure that Web-based interventions are adaptive to the devices preferred by each patient (ie, mobile phone, tablet, or computer) [28,29].

### Strengths and Limitations of the Study

The strengths of the study include adherence to the pilot study protocol and the development of an innovative and complex Web-based tailored intervention.

The limitations of this study mostly relate to pilot study characteristics; the single-group, pre-post study design did not allow for causal inferences or for having enough statistical power to detect statistically significant differences. However, this is expected in a pilot study [45,46]. High dropout rates were observed, particularly in the second and third session of the intervention, leading us to believe that changes must be made to the TAVIE@COEUR Web-based intervention before undertaking an RCT. Finally, we observed in our results that the Morisky SRMTS was not designed for properly quantifying the percentage of medication adherence [38]. Future studies should consider using a different tool or scale for measuring more precisely adherence, such as the medication possession ratio [6,52].

### Conclusions

Web-based tailored interventions such as TAVIE@COEUR show potential to support illness management for patients in all clinical settings. However, significant challenges remain, and future research must be conducted to optimize factors related to the tailoring of such interventions to patients’ knowledge, needs, interests, and individual capabilities. We strongly believe the right combination of these factors could contribute to the way in which care is provided in health care settings for years to come. With continued population acceptance of Internet use, Web-based interventions are expected to grow exponentially in content and in complexity. Health professionals have an important role to play in keeping these interventions focused on patients and their families to optimize clinical outcomes.

## Appendix

Multimedia Appendix 1 CONSORT E-HEALTH Checklist.

## Abbreviations

ACS: acute coronary syndrome
CCU: Coronary Care Unit
CR: cardiac rehabilitation
eHealth: electronic health
HIV: human immunodeficiency virus
ICTs: information and communications technologies
ITS: intelligent tutoring systems
RCT: randomized controlled trial
RN: registered nurse
SD: standard deviation
SRMTS: Self-Reported Medication-Taking Scale
TSCS: Therapeutic Self-Care Scale
